# Growth of Low-Defect Nitrogen-Doped Graphene Film Using Condensation-Assisted Chemical Vapor Deposition Method

**DOI:** 10.3390/ma16031120

**Published:** 2023-01-28

**Authors:** Zhichao Guo, Zhenya Ye, Mengqing Yin, Shixun Dai, Xiaohui Zhang, Wei Wang, Zhaoping Liu

**Affiliations:** 1 School of Information Science and Engineering, Ningbo University, Ningbo 315201, China; 2 Key Laboratory of Graphene Technologies and Applications of Zhejiang Province, CAS Engineering Laboratory for Graphene, Ningbo Institute of Materials Technology & Engineering, Chinese Academy of Sciences, Ningbo 315201, China; 3 Nano Science and Technology Institute, University of Science and Technology of China, Hefei 230026, China; 4 CRRC Industrial Academy Co., Ltd., Beijing 100039, China

**Keywords:** chemical vapor deposition, nitrogen-doped graphene films, condensation-assisted CVD

## Abstract

It is significantly important to modulate the electrical properties of graphene films through doping for building desired electronic devices. One of the effective doping methods is the chemical vapor deposition (CVD) of graphene films with heteroatom doping during the process, but this usually results in nitrogen-doped graphene with low doping levels, high defect density, and low carrier mobility. In this work, we developed a novel condensation-assisted CVD method for the synthesis of high-quality nitrogen-doped graphene (NG) films at low temperatures of 400 °C using solid 3,4,5-trichloropyridine as a carbon and nitrogen source. The condensation system was employed to reduce the volatilization of the solid source during the non-growth stage, which leads to a great improvement of quality of as-prepared NG films. Compared to the one synthesized using conventional CVD methods, the NG films synthesized using condensation-assisted CVD present extremely low defects with a ratio of from D- to G-peak intensity (*I_D_/I_G_*) in the Raman spectrum lower than 0.35. The corresponding total N content, graphitic nitrogen/total nitrogen ratio, and carrier mobility reach 3.2 at%, 67%, and 727 cm^2^V^−1^S^−1^, respectively. This improved condensation-assisted CVD method provides a facile and well-controlled approach for fabricating high-quality NG films, which would be very useful for building electronic devices with high electrical performance.

## 1. Introduction

Graphene has attracted wide attention in the last twenty years owing to its remarkable physical and chemical properties [1,2,3,4,5,6,7], such as high carrier mobility [8], incredible thermal conductivity [9], and excellent optical transparency [10,11]. However, pristine graphene is a zero-bandwidth material, which greatly limits the use of graphene in electronic devices [12,13,14]. Among various doping methods, in situ synthesis techniques using heteroatom doping (e.g., nitrogen, boron, sulfur, and fluorine) are of the most promising methods to adjust the electrical properties of graphene [15,16,17,18]. Doped atoms are added to the graphene lattice to form covalent bonds with C atoms, which will change the electronic structure of graphene and can open the band gap of graphene by suppressing the density of states near the Fermi energy level [19,20,21]. Considering the various heteroatoms used for doping graphene, nitrogen is the most widely used as a dopant due to the similar size between nitrogen and carbon atoms. Substituting nitrogen atoms for carbon atoms will not introduce excessive defects in doped graphene films and hence preserve excellent electrical properties [22], which causes nitrogen-doped graphene to be extremely promising for applications in field-effect transistors [23], electrochemical energy storage [24], and supercapacitors [25].

Many works have been produced mainly on the synthesis of NG films using chemical vapor deposition (CVD) methods using methane or ethane as carbon sources and substances containing nitrogen atoms as the nitrogen dopant, such as methane and ammonia [26] or ethane and acetonitrile [27]. On the other hand, some works have focused on developing a single ingredient containing carbon and nitrogen, such as s-triazine [28], dimethylformamide [29], and melamine [30], which can continuously incorporate carbon and nitrogen elements during the nucleation and growth process of graphene films. These processes usually involve breaking C–C bonds at high reaction temperatures of above 800 °C and then doping nitrogen atoms into the graphene lattice to form C–N bonds [19]. However, NG films synthesized at high temperatures have low doping levels and poor electrical properties, which hinders the practical application of NG films.

Further studies on the fabrication of NG films have been widely investigated at low temperatures. For example, Choi et al. synthesized NG films using laser-induced nitrogen doping of SiC substrates at 600 °C with doping concentration of 0.6 at% [31]. Xue et al. reported a method for the synthesis of NG films using the self-assembly of pyridine molecules at 300 °C, which exhibited n-type doping in the air with carrier mobility in the range of 53.5–72.9 cm^2^V^−1^S^−1^ [32]. Zhang et al. used pentachloropyridine as a precursor to synthesize highly defective NG films at 230 °C using the radical reaction of polyhalogenated aromatic compositions [33]. The CVD synthesis of NG films at low temperatures also has some problems, such as high defect density and low carrier mobility. There have been many attempts to optimize the low-temperature synthesis process to improve the quality of NG films. For example, a two-step method combining low-temperature growth and high-temperature repair processes has been used to improve the quality of synthetic NG films. Cai et al. prepared NG film precursors at low temperature of 350 °C and then annealed the synthesized NG films with the introduction of methane at 1000 °C; the carrier mobility of the synthesized NG films can be improved to 375 cm^2^V^−1^S^−1^ [34]. Wan et al. synthesized NG film using pentachloropyridine precursors at low temperatures and then annealed the precursors at high temperatures of 700–1000 °C to improve the crystalline quality of the NG films [35]. Son M. et al. synthesized large-area continuous NG films by splitting the synthesis process into nucleation and lateral growth stages [36]. Therefore, it is still significant to optimize the synthesis process at low temperatures for obtaining high-quality NG films.

In this work, a novel and facile condensation-assisted CVD method is developed to fabricate high-quality NG films directly at 400 °C without a multi-step post-repair process. The growth process of NG films involves the Ullmann coupling reaction using 3,4,5-trichloropyridine as the precursor. The introduction of continuous condensation in the carbon and nitrogen source regions during the non-growth stage is beneficial for the controllable preparation of NG films. Finally, the NG films with extremely low defects are obtained, which present excellent electrical properties.

## 2. Materials and Methods

### 2.1. Synthesis of NG Films

**Figure 1:**
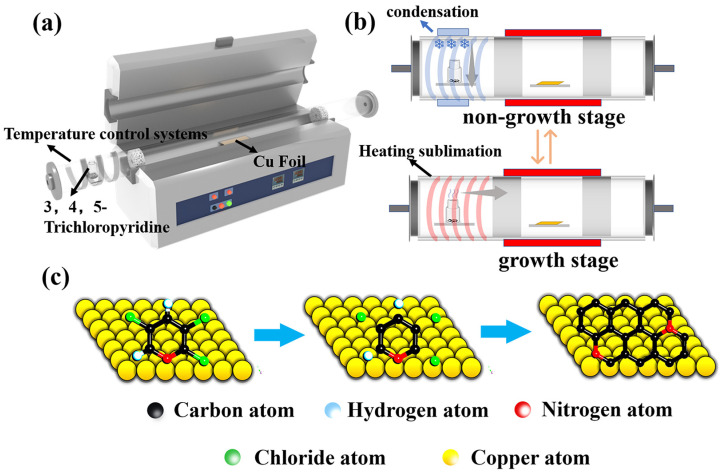
(**a**) Schematic diagram of preparing NG films on copper foil using 3,4,5-trichloropyridine as the source of carbon and nitrogen. (**b**) Diagram of the solid source region at different stages of synthesis in scheme II. (**c**) Growth of NG films on the copper foil via Ullmann radical coupling mechanism.

The copper foils (Alfa Aesar, 25 μm thick, 99.9% purity, polycrystalline copper) cleaned with glacial acetic acid and ethanol are used as the catalytic substrate. Before the reaction, the Cu foil was placed in the center of the hot-wall CVD quartz tube with a diameter of 13 cm. The 3,4,5-trichloropyridine (Aladdin, 98%) was placed upstream of the CVD system as the carbon and nitrogen source to synthesize NG films. Then, the tube furnace chamber was evacuated to reach the pressure of 80 Pa from the ambient pressure. After switching off the vacuum pump, the furnace chamber was cleaned several times with argon to ensure the interference of residual air was minimized. To remove the oxide layer and improve the surface morphology of the copper foil, the copper foil was annealed at 1000 °C for 20 min under a continuous flow of hydrogen gas of 120 standard cubic centimeters per minute (sccm) at a pressure of 200 Pa and then cooled to the growth temperature of 400–800 °C within 1 h. Here, we used two experimental schemes. Scheme I: After reaching the synthesis temperature, the 3,4,5-trichloropyridine was heated using a heating device to the gaseous stage as the source of carbon and nitrogen, which was then introduced into the growth zone to grow the NG films at an ambient pressure of 200 Pa (Figure 1a). The H_2_ in flow rate of 40 sccm and Ar in the flow rate of 40 sccm were introduced for 0.5–3 min, respectively. After that, the CVD system was kept cooled to room temperature in the hydrogen and argon gas streams (5 sccm and 100 sccm, respectively). Scheme II: A similar process was used for the growth of NG films. Different from scheme I, the 3,4,5-trichloropyridine placement area was continuously cooled using a condensing device during the non-growth phase (including the ramping step before the growth process and cooling step after the growth process) to maintain the temperature of the zone at 10–15 °C (Figure 1b and Figure S1). The evaporation of 3,4,5-trichloropyridine can be eliminated during the non-growth step (Figure S2). At the same time, condensation was switched on at different time intervals in the solid source region at the end of growth to investigate the effect of the condensation start time on the quality of the synthesized graphene films.

### 2.2. Synthesis of Pristine Graphene Films

The pristine graphene films were synthesized using the conventional CVD method by placing the copper foil in a quartz tube and heating it to 1000 °C in an atmosphere of hydrogen, where the hydrogen rates were 100 sccm, and then introducing 40 sccm of methane for 30 min to grow graphene films on the surface of the copper foil at an ambient pressure of 200 Pa. Finally, the growth area was cooled to room temperature.

### 2.3. Transfer Process of NG Films

The synthesized NG films were transferred onto 300 nm SiO_2_/Si substrate by using a poly methyl methacrylate (PMMA, Alfa, 20 mg/mL dissolved in ethyl acetate)-assisted transferring method. Briefly, the PMMA precursor was spin-coated on the surface of the graphene/Cu and then cured at 90 °C for 15 min to solidify the polymer. Subsequently, the sample was suspended in FeCl_3_ aqueous solution (0.8 mol/L) to etch away the copper film. The floating PMMA/Graphene films were scooped up using clean silicon wafers and washed using deionized water repeatedly. Finally, an acetone was used to remove the PMMA layer.

### 2.4. Characterization

The NG films and pristine films were characterized using Raman spectroscopy (Renishaw inVia Reflex with laser excitation energy of 532 nm, Renishaw, London, UK), Cold field emission scanning electron microscopy (SEM, S4800, 4 kV, Hitachi, Tokyo, Japan), X-ray photoelectron spectroscopy (XPS, AXIS SUPRA, Kratos, Manchester, UK), Ultra-violet photoemission spectroscopy (UPS, Axis Ultra DLD, Kratos, Manchester, UK), Polarized optical microscopy (POM, BX51, OLYMPUS, Tokyo, Japan), and The Hall Effect Measurement System (8404-CRX-6.5K, Lake Shore, Columbus, OH, USA).

## 3. Results and Discussion

Figure 1a presents the typical experiment setup. It is worth mentioning that heating or cooling the 3,4,5-trichloropyridine as the carbon and nitrogen source is in the upstream place and NG films would be grown on the center area in the CVD system. As shown in Figure 1b and Figure S1, in scheme II, the condensing device is used at the periphery of the 3,4,5-trichloropyridine placement zone to suppress the evaporation of the solid carbon and nitrogen source during the non-growth stages, including warming, annealing, and cooling stages. As shown in Figure 1c, the growth process of NG films follows the Ullmann reaction [37]. The halogenated aromatic hydrocarbons are heated in the presence of copper foils to generate the bottom-up self-assembly reaction and form biphenyl compounds using dichlorination and dehydrogenation processes. Finally, it facilitates the formation of the graphene network structure with substituted nitrogen atoms.

**Figure 2:**
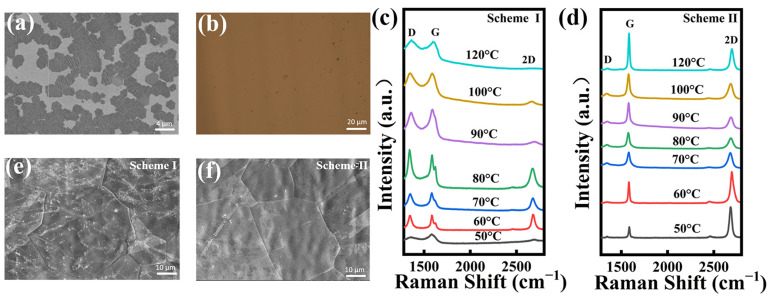
(**a**) SEM images of NG films on the copper surface after CVD growth for 0.5 min. (**b**) Optical image of a typical NG film after CVD growth for three minutes transferred to SiO_2_/Si. Raman spectra of NG films synthesized at different sublimation temperatures in the solid source region using (**c**) scheme I and (**d**) scheme II, respectively. SEM images of NG films grown for 3 min using (**e**) scheme I and (**f**) scheme II, respectively.

The SEM image of the NG film grown at 400 °C for 0.5 min is shown in Figure 2a. Mass graphene domains formed on the copper foil surface within a short time. Figure 2b presents the optical image of the NG film grown for three minutes transferred onto a SiO_2_/Si substrate that exhibits good coverage and uniform surface. Figure 2c,d present Raman spectra of NG films synthesized at 400 °C by controlling the solid source region at different sublimation temperatures using scheme I and scheme II, respectively. The characteristic peaks of graphene are shown in Raman spectra: D-peak, G-peak, and 2D-peak. It is known that the D band, a first-order zone boundary phonon mode related to graphene edge and dangling bonds, represents the quality of graphene films [38,39]. As seen in Figure 2c, the presence of a distinct G-peak and a broad 2D-peak in the NG film indicates that the graphene synthesized using scheme I has significant defects. Additionally, as the sublimation temperature increased, a large amount of the solid source was introduced into the growth region and adsorbed on the surface of the copper foil, which affected the nucleation growth of graphene, resulting in the synthesized graphene films having poor crystalline quality and multilayer structures.

Compared to scheme I, the D peaks of all the NG films fabricated at different sublimation temperatures using scheme II can be negligible, as shown in Figure 2d. These phenomena demonstrate that the defects in NG films are greatly affected by the control of volatilization of 3,4,5-trichloropyridine. It suggests that 3,4,5-trichloropyridine might still volatilize due to heat spreading from the growth region and further leads to the uncontrollable growth of NG films during the non-growth stages of NG films (warming, annealing, and cooling stages). Therefore, the use of a condensation system in the solid source placement area and the control of the temperature in the solid source area during the non-growth stage could significantly eliminate the unexpected volatilization of the solid source and finally facilitate the synthesis of high-quality NG films.

The ratio of the 2D peak to the G-peak intensity (*I_2D_/I_G_*) is an important role in indicating the layers of graphene films. It is known that the value of *I_2D_/I_G_* decreases as the number of graphene layers increases [40,41]. The Raman spectra of the NG films synthesized using scheme I reveal the nature of multiple layers with *I_2D_/I_G_* < 1. However, the NG films synthesized using scheme II are monolayers with *I_2D_/I_G_* > 1.5 at sublimation temperatures of 40 °C and 50 °C. Thus, the low-defect monolayer NG films can be realized at these optimal fabrication parameters. Figure 2e shows the SEM images of NG films grown for three minutes using scheme I. The continuous NG film has been formed to cover the whole surface of the copper foil within three minutes. Meanwhile, a large number of white impurities can be observed on the surface over the whole sample grown using scheme I. It becomes very clean for the NG films grown using scheme II as shown in Figure 2f. The results indicate that the introduction of condensation systems in scheme II can substantially reduce the uncontrollable volatilization of the solid source during the non-growth stage and hence significantly improve the quality of NG films.

**Figure 3:**
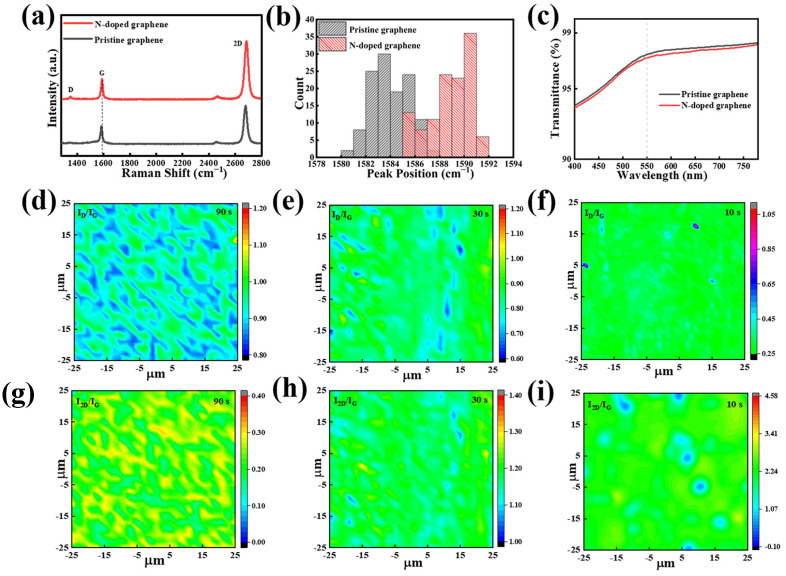
(**a**) Raman spectra of the NG films and pristine graphene films. (**b**) Distribution chart of Raman G-peak position of pristine graphene and NG films. (**c**) The transmittance of the pristine graphene and NG films. Spatial maps of (**d**–**f**) *I_D_/I_G_* and (**g**–**i**) *I_2D_/I_G_* of the NG films using condensation treatment starting at 90 s, 30 s, and 10 s after the end of growth, respectively. D and G represent the peaks of the curve.

Further studies were carried out on NG films synthesized using scheme II and grown at a sublimation temperature of 50 °C for 3 min. As shown in Figure 3a, the Raman spectrum of the NG film exhibit characteristic peaks at 1347.3 cm^−1^, 1588.3 cm^−1^, and 2684.4 cm^−1^, which correspond to the D, G, and 2D peaks, respectively [42,43]. Compared to the pristine graphene films prepared using the conventional CVD process at high temperature, the Raman spectrum of the NG films exhibits a slightly higher D peak, which might be due to structural defects caused by the nitrogen doping. Further detailed observation reveals the blue shift of the G peaks in the Raman spectra. Figure 3b compares the statistical counts of the G peaks of the NG films and pristine films in different areas. It clearly shows that the G peaks in the Raman spectra of NG films have higher wave numbers compared to that of the pristine graphene films. Since the nitrogen atom is substitutionally incorporated into the graphene lattice, it will generate point defects in the sp^2^ lattice [44,45]. Therefore, the in-plane grain dimension can be calculated using the new formula provided by Ferrari et al. instead of the conventional Tuinstra–Koening formula [46]:

$$L_{D}^{2}{\left(\mathrm{nm}\right)}^{2}=(1.8\pm 0.5)\times 10^{-9}\lambda_{L}^{4}{\left(\frac{I_{D}}{I_{G}}\right)}^{-1}$$

The length dimension (*L_D_*) drops from 21.8–42.9 nm for the pristine graphene films to 15.8–18.2 nm for the NG films, which is consistent with the high doping level in the NG films. As shown in Figure 3c, the transmittance measured at 550 nm for the NG films is approximately 97.3%, which is similar to the value of 97.5% for the pristine graphene film.

Raman spectra mapping is also measured to evaluate the defects, the number of layers, and the uniformity of the NG films. To reduce inaccuracy, the area in size of 50 μm × 50 μm was chosen for the Raman mapping. Figure 3d–f show the *I_D_/I_G_* mapping of the NG films obtained by starting the condensation system at different times of 90 s, 30 s, and 10 s after the end of CVD growth, respectively. It can be seen that the distribution of *I_D_/I_G_* becomes more uniform as the time interval decreases and also that the *I_D_/I_G_* decreases from 0.85–0.98 to 0.25–0.45 due to the reduction in the on-condensation time interval from 90 s to 10 s (Figure S3a). The result demonstrates that starting the condensation system at shorter time intervals after the end of growth can lead to the formation of uniform and low-defect NG films. Meanwhile, Figure 3g–i and Figure S3b show the corresponding Raman mapping of *I_2D_/I_G_*. Similarly, the value of *I_2D_/I_G_* becomes higher and more uniform when decreasing the condensation starting time. It indicates that starting the condensation system at a shorter time after the end of CVD growth is beneficial for the synthesis of homogeneous monolayer NG films, which might be attributed to the effective termination of volatilization of the solid source during the non-growth stage.

**Figure 4:**
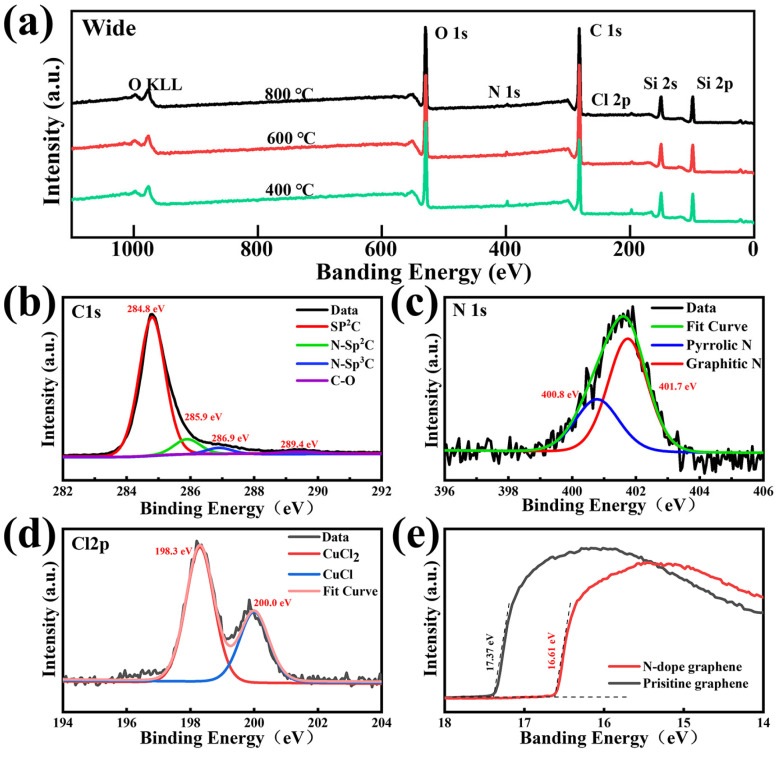
(**a**) XPS spectra of the NG films grown at different temperatures of 400 °C, 600 °C, and 800 °C, respectively. High-resolution XPS spectra of (**b**) C 1s, (**c**) N 1s, and (**d**) Cl 2p peaks of the corresponding NG films. (**e**) The graph of the work function of pristine graphene and NG films.

The XPS spectra of the NG films on the SiO_2_/Si substrate are measured in detail to evaluate the N content and bonding structure. Figure 4a shows the levels of C 1s, O 1s, Cl 2p, and N1s of the NG films fabricated at different temperatures of 400 °C, 600 °C, and 800 °C, respectively. The signal of N 1s increases slightly when decreasing the synthesis temperature. The total nitrogen concentration increases from 1.6 at% to 3.2 at% by decreasing the synthesis temperature from 800 °C to 400 °C, which indicates that the nitrogen doping degree of the NG films is negatively related to the synthesis temperature. The peak at 284.8 eV is attributed to graphite-like carbon atoms, whereas the peaks at 285.9 eV, 286.9 eV, and 289.4 eV are related to various C–N bonding components, including sp^2^- and sp^3^- hybridized carbon atoms and the C–O bonding type [35], as shown in Figure 4b. In the doping process, the nitrogen atoms are added to the graphene lattice in three main ways: pyridine N, pyrrole N, and graphite N. Pyridine N is a nitrogen atom that forms a covalent bond with two adjacent carbon atoms in the six-membered ring at the edge or defect position of graphene; pyrrole N is a nitrogen atom that forms a covalent bond with two adjacent carbon atoms in the five-membered ring; and graphite N is a nitrogen atom that directly replaces a carbon atom in the six-membered ring [47]. Among the above three nitrogen forms, the formation of pyridine and pyrrole nitrogen introduces defects and breaks the symmetry of the graphene lattice [30]. Figure 4c presents the high-resolution XPS spectrum of N 1s, which reveals two forms of C–N bonding that pyrrolic N is centered at 400.8 eV and graphitic N is centered at 401.7 eV, respectively [48]. The absence of a significant pyridine nitrogen signal indicates that the nitrogen atoms can be incorporated into the graphene lattice as pyrrole and graphite nitrogen at low temperatures. It is worth mentioning that the NG films prepared at 400 °C reflect a higher level of graphitic nitrogen signal compared to that of the NG films prepared at 600 °C and 800 °C, as shown in Figure S4. The ratio between the graphitic-N content and total N content reached 67%, indicating that the graphitic nitrogen structure is the main form of nitrogen atoms incorporated into the graphene lattice. The overall nitrogen doping content and graphitic nitrogen content decreased as the synthesis temperature increased. The higher synthesis temperatures lead to more breaking of the carbon and nitrogen bonds, which causes it to be more difficult to form graphitic nitrogen structures [19,30]. The total nitrogen doping content decreases when increasing the synthesis temperature, which is consistent with the result in Figure 4a. It can be also found that the carbon–nitrogen bonding structure of the as-grown NG films at 800 °C is dominated by pyrrole nitrogen, which demonstrates that pyrrole nitrogen has a higher thermal stability than pyridine and graphite nitrogen. In detail, as shown in Figure 4d, the asymmetric Cl 2p peak can be split into two components of around 198.3 eV and 200.0 eV, which corresponds to the signal of CuCl and CuCl_2_ [49,50]. This result indicates that the chlorine atoms in 3,4,5-trichloropyridine would be more likely to react with the copper substrate to form copper chloride and cuprous chloride rather than be added to the grid of carbon atoms. Figure 4e shows the work function of the NG films and pristine graphene films. Compared with the pristine graphene films, the work function of the synthesized NG films sample is reduced by 0.76 eV, this is because, during the doping process, nitrogen atoms are added to the graphene lattice as electron donors and the Fermi energy level of graphene is shifted, resulting in a decrease in the work function [51]. Therefore, NG films exhibit n-type doping properties.

**Figure 5:**
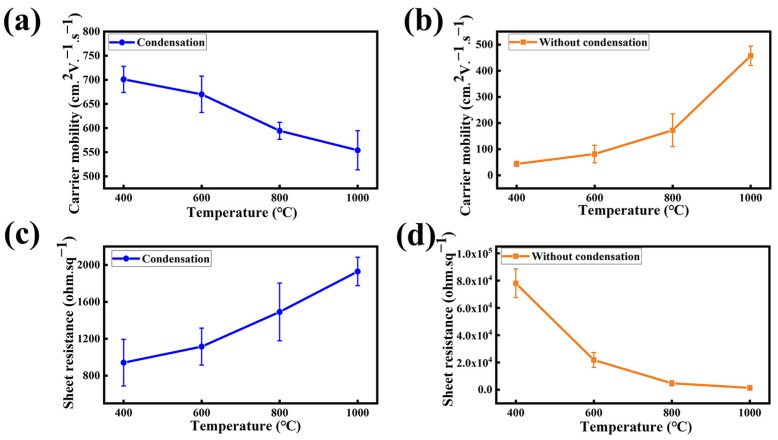
Graph of the carrier mobility of NG films at different synthesis temperatures of 400 °C, 600 °C, 800 °C, and 1000 °C, (**a**) with using condensation treatment and (**b**) without using condensation treatment. Graph of the sheet resistance of NG films at different synthesis temperatures of 400 °C, 600 °C, 800 °C, and 1000 °C, (**c**) with using condensation treatment and (**d**) without using condensation treatment.

Furthermore, a Hall effect measurement has been carried out on the synthesized NG films on SiO_2_/Si to characterize the corresponding electrical properties. The carrier mobility distribution of the NG films grown at the synthesis temperatures of 400 °C, 600 °C, 800 °C, and 1000 °C is shown in Figure 5a. For the NG film grown at the low temperature of 400 °C, the carrier mobility of the NG film reaches an optimal value of 727 cm^2^V^−1^s^−1^, which is better than previously reported (Table 1). When increasing the synthesis temperature to 1000 °C, the corresponding carrier mobility decreases to 602 cm^2^V^−1^s^−1^. The drop of the carrier mobility with the increasing synthesis temperature may be related to its reaction mechanism. In a low-temperature environment, the formation of graphene lattices depends mainly on the self-assembly coupling mechanism. Chemical bond is impelled to break and rearrange in the high-temperature environment and, hence, leads to the formation of more defects. Compared to the NG films grown using the condensation treatment, the NG films fabricated without using condensation treatment exhibit lower carrier mobility, as shown in Figure 5b. Moreover, the carrier mobility of the NG films fabricated without using condensation treatment exhibits a positive temperature coefficient. This is probably because the lack of condensation treatment allowed the solid source to volatilize early to form precursors and then the high-temperature annealing optimized the quality of the NG films [35]. 

Figure 5c,d show the cube resistance corresponding to the carrier mobility, with the NG films using the condensation treatment demonstrating a lower sheet resistance compared to the NG films synthesized without the condensation treatment. The result demonstrates that the condensation-assisted process can significantly reduce the graphene defects and effectively improve the electrical properties of the NG films by suppressing the excessive volatilization of the solid source.

## 4. Conclusions

In summary, a novel and facile condensation-assisted CVD method was successfully developed to synthesize low-defect NG films at 400 °C using 3,4,5-trichloropyridine as the sole solid source. Compared to the NG films fabricated using the conventional CVD process, the NG films fabricated with the help of condensation treatment on the solid source during the non-growth stage exhibit much lower defect signal of *I_D_* in Raman spectra and uniform monolayer structure in large area. The result suggests that the introduction of a condensation system could avoid the excessive volatilization of solid sources and hence preserve NG films from the formation of defects and impurities. Finally, the NG films prepared at an optimal temperature of 400 °C using a condensation-assisted device exhibit an extremely high graphitic-N/total N percentage of 67%, low defect of *I_D_/I_G_* < 0.4, and excellent carrier mobility of 727 cm^2^V^−1^S^−1^. This work provides an effective approach to grow high-quality NG films using solid sources that could lead to the great improvement in the electrical applications of NG films.

## Figures and Tables

**Table 1:** Carrier mobility of NG films.

N Source	C Source	Growing Temperature	Mobility (cm^2^V^−1^S^−1^)	Ref.
Ammonia	Methane	800 °C	200–450	[26]
S-triazine	-	700 °C	11.7	[28]
Dimethylformamide	-	950 °C	310–630	[29]
Melamine	-	1000 °C	74	[30]
Pyridine	-	300 °C	53.5–72.9	[32]
Pentachloropyridine	-	230 °C	80.1–302.7	[33]
Pentachloropyridine	Methane	350 °C	375	[34]
Pentachloropyridine	-	400–600 °C	522	[35]
3,4,5-trichloropyridine	-	400 °C	727	This work

## Data Availability

All data that support the findings of this study are included within the article and Supplementary Materials.

## Supplementary Materials

The following supporting information can be downloaded at: https://www.mdpi.com/article/10.3390/ma16031120/s1, Figure S1: Scheme I: (a) non-growth and (b) growth stage. Scheme II: (c) Heating up and annealing phases, (d) Growth stage, and (e) Cooling stage; Figure S2: 3,4,5-trichloropyridine deposited on the inner wall of quartz tube by using condensation system; Figure S3: Raman mapping of NG films prepared by condensing the carbon and nitrogen source regions at different time intervals (90 s, 30 s, and 10 s) after the end of growth: (a) Ratio of D-peak to G-peak intensity (*I_D_/I_G_*), (b) Ratio of 2D peak to G-peak intensity (*I_2D_/I_G_*); Figure S4: High resolution N1s peaks collected from 600 °C (a) and 800 °C (b) grown NG films.

## References

[1] Geim A.K., Novoselov K.S. (2007). The rise of graphene. Nat. Mater..

[2] Lee C., Wei X.D., Kysar J.W., Hone J. (2008). Measurement of the elastic properties and intrinsic strength of monolayer graphene. Science.

[3] Castro Neto A.H., Guinea F., Peres N.M.R., Novoselov K.S., Geim A.K. (2009). The electronic properties of graphene. Rev. Mod. Phys..

[4] Geim A.K. (2009). Graphene: Status and Prospects. Science.

[5] Allen M.J., Tung V.C., Kaner R.B. (2010). Honeycomb Carbon: A Review of Graphene. Chem. Rev..

[6] Neuville S., Matthews A. (2007). A perspective on the optimisation of hard carbon and related coatings for engineering applications. Thin Solid Films.

[7] Keshri A.K., Agarwal A. (2011). Splat morphology of plasma sprayed aluminum oxide reinforced with carbon nanotubes: A comparison between experiments and simulation. Surf. Coat. Technol..

[8] Bolotin K.I., Sikes K.J., Jiang Z., Klima M., Fudenberg G., Hone J., Kim P., Stormer H.L. (2008). Ultrahigh electron mobility in suspended graphene. Solid State Commun..

[9] Kim K.S., Zhao Y., Jang H., Lee S.Y., Kim J.M., Kim K.S., Ahn J.H., Kim P., Choi J.Y., Hong B.H. (2009). Large-scale pattern growth of graphene films for stretchable transparent electrodes. Nature.

[10] Bonaccorso F., Sun Z., Hasan T., Ferrari A.C. (2010). Graphene photonics and optoelectronics. Nat. Photonics.

[11] Wang X., Zhi L.J., Mullen K. (2008). Transparent, conductive graphene electrodes for dye-sensitized solar cells. Nano Lett..

[12] Son Y.W., Cohen M.L., Louie S.G. (2006). Half-metallic graphene nanoribbons. Nature.

[13] Han M.Y., Ozyilmaz B., Zhang Y.B., Kim P. (2007). Energy band-gap engineering of graphene nanoribbons. Phys. Rev. Lett..

[14] Schwierz F. (2010). Graphene transistors. Nat. Nanotechnol..

[15] Altuntepe A., Zan R. (2021). Permanent Boron Doped Graphene with high Homogeneity using Phenylboronic Acid. J. Mol. Struct..

[16] Zhou J.H., Wang Z.G., Chen Y.F., Liu J.B., Zheng B.J., Zhan W.L., Li Y.R. (2017). Growth and properties of large-area sulfur-doped graphene films. J. Mater. Chem. C.

[17] Yang Z., Yao Z., Li G.F., Fang G.Y., Nie H.G., Liu Z., Zhou X.M., Chen X., Huang S.M. (2012). Sulfur-Doped Graphene as an Efficient Metal-free Cathode Catalyst for Oxygen Reduction. ACS Nano.

[18] Shen B.S., Chen J.T., Yan X.B., Xue Q.J. (2012). Synthesis of fluorine-doped multi-layered graphene sheets by arc-discharge. RSC Adv..

[19] Li X.L., Wang H.L., Robinson J.T., Sanchez H., Diankov G., Dai H.J. (2009). Simultaneous Nitrogen Doping and Reduction of Graphene Oxide. J. Am. Chem. Soc..

[20] Gueorguiev G.K., Neidhardt J., Stafstrom S., Hultman L. (2005). First-principles calculations on the curvature evolution and cross-linkage in carbon nitride. Chem. Phys. Lett..

[21] Gueorguiev G.K., Neidhardt J., Stafstrom S., Hultman L. (2005). First-principles calculations on the role of CN precursors for the formation of fullerene-like carbon nitride. Chem. Phys. Lett..

[22] Deokar G., Jin J., Schwingenschlogl U., Costa P. (2022). Chemical vapor deposition-grown nitrogen-doped graphene’s synthesis, characterization and applications. NPJ 2D Mater. Appl..

[23] Guo B.D., Liu Q.A., Chen E.D., Zhu H.W., Fang L.A., Gong J.R. (2010). Controllable N-Doping of Graphene. Nano Lett..

[24] Wen Y.Y., Huang C.C., Wang L.Z., Hulicova-Jurcakova D. (2014). Heteroatom-doped graphene for electrochemical energy storage. Chin. Sci. Bull..

[25] Deng Y.F., Xie Y., Zou K.X., Ji X.L. (2016). Review on recent advances in nitrogen-doped carbons: Preparations and applications in supercapacitors. J. Mater. Chem. A.

[26] Wei D.C., Liu Y.Q., Wang Y., Zhang H.L., Huang L.P., Yu G. (2009). Synthesis of N-Doped Graphene by Chemical Vapor Deposition and Its Electrical Properties. Nano Lett..

[27] Reddy A.L.M., Srivastava A., Gowda S.R., Gullapalli H., Dubey M., Ajayan P.M. (2010). Synthesis Of Nitrogen-Doped Graphene Films For Lithium Battery Application. ACS Nano.

[28] Lu Y.F., Lo S.T., Lin J.C., Zhang W.J., Lu J.Y., Liu F.H., Tseng C.M., Lee Y.H., Liang C.T., Li L.J. (2013). Nitrogen-Doped Graphene Sheets Grown by Chemical Vapor Deposition: Synthesis and Influence of Nitrogen Impurities on Carrier Transport. ACS Nano.

[29] Gao H., Song L., Guo W.H., Huang L., Yang D.Z., Wang F.C., Zuo Y.L., Fan X.L., Liu Z., Gao W. (2012). A simple method to synthesize continuous large area nitrogen-doped graphene. Carbon.

[30] Wang Z.G., Li P.J., Chen Y.F., Liu J.B., Tian H.J., Zhou J.H., Zhang W.L., Li Y.R. (2014). Synthesis of nitrogen-doped graphene by chemical vapour deposition using melamine as the sole solid source of carbon and nitrogen. J. Mater. Chem. C.

[31] Choi I., Jeong H.Y., Jung D.Y., Byun M., Choi C.G., Hong B.H., Choi S.Y., Lee K.J. (2014). Laser-Induced Solid-Phase Doped Graphene. ACS Nano.

[32] Xue Y.Z., Wu B., Jiang L., Guo Y.L., Huang L.P., Chen J.Y., Tan J.H., Geng D.C., Luo B.R., Hu W.P. (2012). Low Temperature Growth of Highly Nitrogen-Doped Single Crystal Graphene Arrays by Chemical Vapor Deposition. J. Am. Chem. Soc..

[33] Zhang J., Li J.J., Wang Z.L., Wang X.N., Feng W., Zheng W., Cao W.W., Hu P.A. (2014). Low-Temperature Growth of Large-Area Heteroatom-Doped Graphene Film. Chem. Mater..

[34] Cai W., Wang C., Fang X.H., Yang L.Y., Chen X.Y. (2015). Synthesis and characterization of nitrogen-doped graphene films using C_5_NCl_5_. Appl. Phys. Lett..

[35] Wan J.X., You Y., Xu Y.L., Wang C., Zhang P.B., Jiang X.Y., Fang X.H., Yang L.Y., Chen X.Y. (2017). Synthesis of nitrogen-doped graphene via pentachloropyridine as the sole solid source. Appl. Phys. Lett..

[36] Son M., Chee S.S., Kim S.Y., Lee W., Kim Y.H., Oh B.Y., Hwang J.Y., Lee B.H., Ham M.H. (2020). High-quality nitrogen-doped graphene films synthesized from pyridine via two-step chemical vapor deposition. Carbon.

[37] Ullmann F. (1903). Ueber eine neue Bildungsweise von Diphenylaminderivaten. Chem.Ges..

[38] Malard L.M., Pimenta M.A., Dresselhaus G., Dresselhaus M.S. (2009). Raman spectroscopy in graphene. Phys. Rep..

[39] Eckmann A., Felten A., Mishchenko A., Britnell L., Krupke R., Novoselov K.S., Casiraghi C. (2012). Probing the Nature of Defects in Graphene by Raman Spectroscopy. Nano Lett..

[40] Ferrari A.C., Meyer J.C., Scardaci V., Casiraghi C., Lazzeri M., Mauri F., Piscanec S., Jiang D., Novoselov K.S., Roth S. (2006). Raman spectrum of graphene and graphene layers. Phys. Rev. Lett..

[41] Silva D.L., Campos J.L.E., Fernandes T.F.D., Rocha J.N., Machado L.R.P., Soares E.M., Miquita D.R., Miranda H., Rabelo C., Neto O.P.V. (2020). Raman spectroscopy analysis of number of layers in mass-produced graphene flakes. Carbon.

[42] Ferrari A.C. (2007). Raman spectroscopy of graphene and graphite: Disorder, electron-phonon coupling, doping and nonadiabatic effects. Solid State Commun..

[43] Ferrari A.C., Basko D.M. (2013). Raman spectroscopy as a versatile tool for studying the properties of graphene. Nat. Nanotechnol..

[44] Zafar Z., Ni Z.H., Wu X., Shi Z.X., Nan H.Y., Bai J., Sun L.T. (2013). Evolution of Raman spectra in nitrogen doped graphene. Carbon.

[45] Das A., Pisana S., Chakraborty B., Piscanec S., Saha S.K., Waghmare U.V., Novoselov K.S., Krishnamurthy H.R., Geim A.K., Ferrari A.C. (2008). Monitoring dopants by Raman scattering in an electrochemically top-gated graphene transistor. Nat. Nanotechnol..

[46] Cancado L.G., Jorio A., Ferreira E.H.M., Stavale F., Achete C.A., Capaz R.B., Moutinho M.V.O., Lombardo A., Kulmala T.S., Ferrari A.C. (2011). Quantifying Defects in Graphene via Raman Spectroscopy at Different Excitation Energies. Nano Lett..

[47] Hu Z.N., Zhao Y.X., Zou W.T., Lu Q., Liao J.H., Li F.F., Shang M.P., Lin L., Liu Z.F. (2022). Doping of Graphene Films: Open the way to Applications in Electronics and Optoelectronics. Adv. Funct. Mater..

[48] Zhang C.H., Fu L., Liu N., Liu M.H., Wang Y.Y., Liu Z.F. (2011). Synthesis of Nitrogen-Doped Graphene Using Embedded Carbon and Nitrogen Sources. Adv. Mater..

[49] Kishi K., Ikeda S. (1974). X-ray Photoelectron spectroscopic study of reaction of evaporated metal-films with chlorine gas. J. Phys. Chem..

[50] Ito Y., Christodoulou C., Nardi M.V., Koch N., Klaui M., Sachdev H., Mullen K. (2015). Tuning the Magnetic Properties of Carbon by Nitrogen Doping of Its Graphene Domains. J. Am. Chem. Soc..

[51] Deifallah M., McMillan P.F., Cora F. (2008). Electronic and structural properties of two-dimensional carbon nitride graphenes. J. Phys. Chem. C.

