# Supplementary material for: Growth of Low-Defect Nitrogen-Doped Graphene Film Using Condensation-Assisted Chemical Vapor Deposition Method
Source: Materials (Basel). 2023 Jan 28;16(3):1120. doi: 10.3390/ma16031120 (PMC9921703; doi:10.3390/ma16031120)
Supplement: Supplementary file 1 [file materials-16-01120-s001.zip › materials-2173581-SI.pdf]

materials

Article

Growth of Low-Defect Nitrogen-Doped Graphene Film Using Condensation-Assisted Chemical Vapor Deposition Method

Zhichao Guo [1,2], Zhenya Ye [2,3], Mengqing Yin [1,2], Shixun Dai [1], Xiaohui Zhang [4], Wei Wang [2,*] and Zhaoping Liu [2,*]

1 School of Information Science and Engineering, Ningbo University, Ningbo 315201, China
2 Key Laboratory of Graphene Technologies and Applications of Zhejiang Province, CAS Engineering Laboratory for Graphene, Ningbo Institute of Materials Technology & Engineering, Chinese Academy of Sciences, Ningbo 315201, China
3 Nano Science and Technology Institute, University of Science and Technology of China, Hefei 230026, China
4 CRRC Industrial Academy Co., Ltd., Beijing 100039, China
* Correspondence: wangwei@nimte.ac.cn (W.W.); liuzp@nimte.ac.cn (Z.L.)

Supporting information

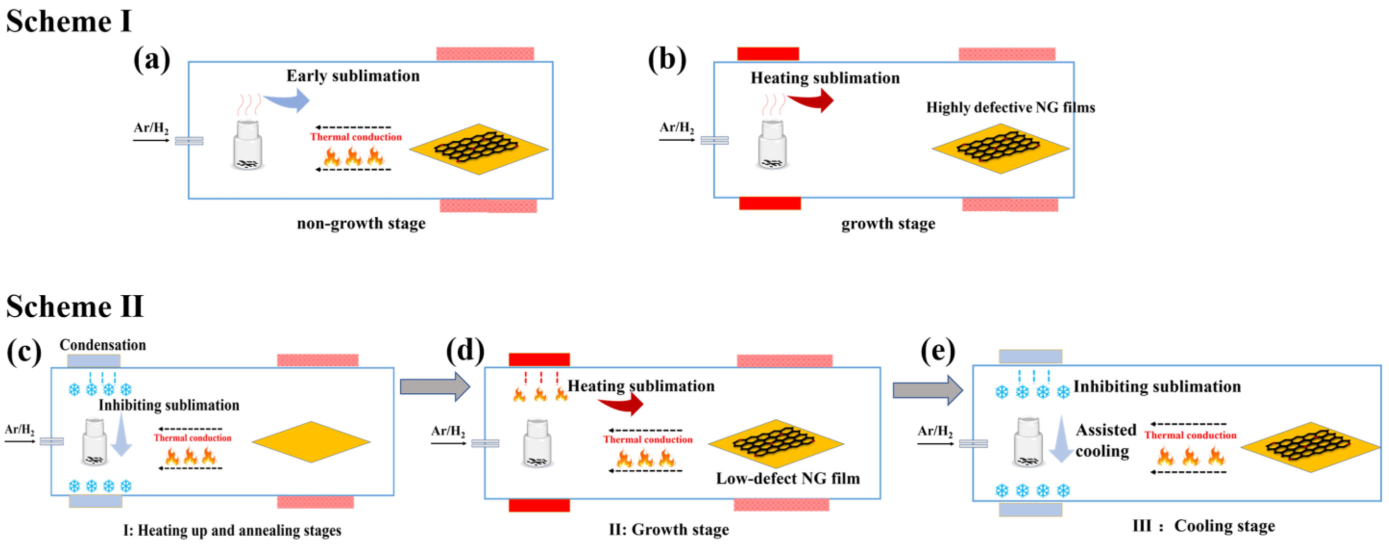

Figure S1. Scheme I: (a)non-growth and (b) growth stage. Scheme II: (c) Heating up and annealing phases, (d) Growth stage, (e) Cooling stage.

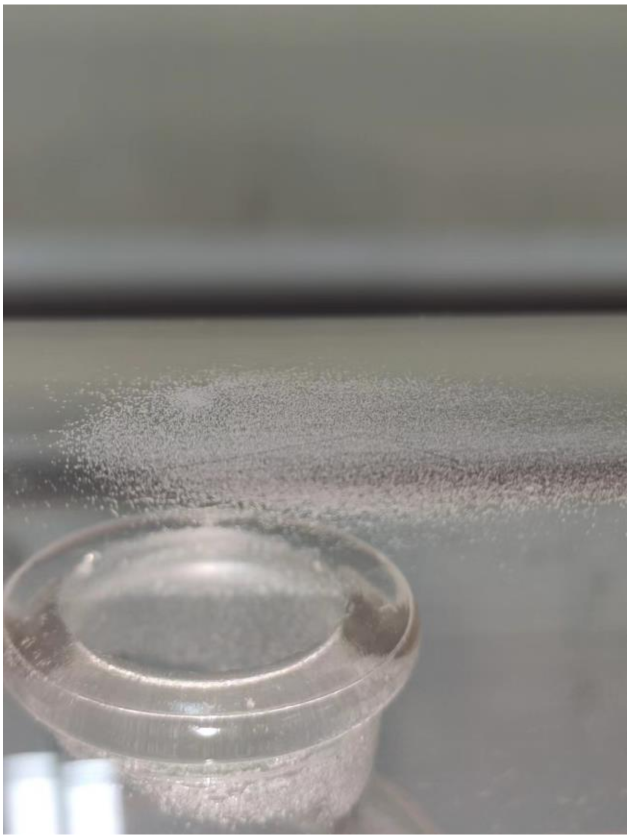

Figure S2. 3,4,5-trichloropyridine deposited on the inner wall of quartz tube by using condensation system.

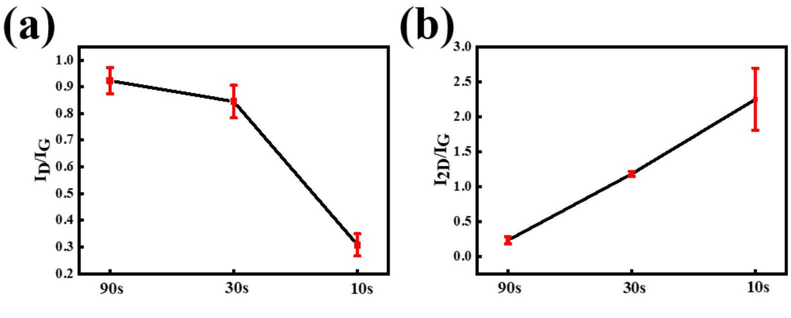

Figure S3. Raman mapping of NG films prepared by condensing the carbon and nitrogen source regions at different time intervals (the 90 s, 30 s, 10 s) after the end of growth:(a) Ratio of D-peak to G-peak intensity (I_D/I_G), (b) Ratio of 2D peak to G peak intensity (I_{2D}/I_G)

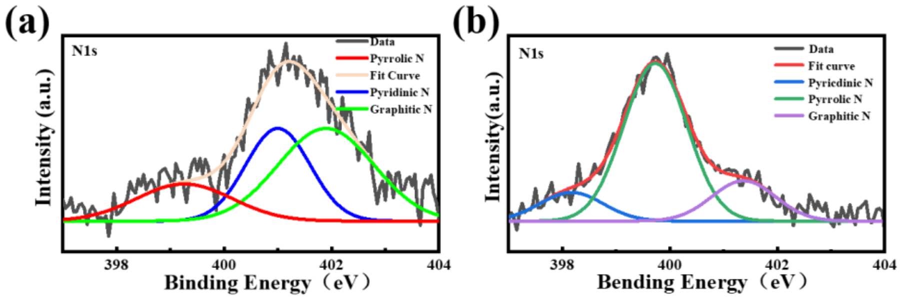

Figure S4. High resolution N1s peaks collected from 600 °C(a) and 800 °C(b) grown NG films
